# Metabolic Causes of Epileptic Encephalopathy

**DOI:** 10.1155/2013/124934

**Published:** 2013-05-22

**Authors:** Joe Yuezhou Yu, Phillip L. Pearl

**Affiliations:** Department of Neurology, Children's National Medical Center, 111 Michigan Avnue, Washington, DC 20010, USA

## Abstract

Epileptic encephalopathy can be induced by inborn metabolic defects that may be rare individually but in aggregate represent a substantial clinical portion of child neurology. These may present with various epilepsy phenotypes including refractory neonatal seizures, early myoclonic encephalopathy, early infantile epileptic encephalopathy, infantile spasms, and generalized epilepsies which in particular include myoclonic seizures. There are varying degrees of treatability, but the outcome if untreated can often be catastrophic. The importance of early recognition cannot be overemphasized. This paper provides an overview of inborn metabolic errors associated with persistent brain disturbances due to highly active clinical or electrographic ictal activity. Selected diseases are organized by the defective molecule or mechanism and categorized as small molecule disorders (involving amino and organic acids, fatty acids, neurotransmitters, urea cycle, vitamers and cofactors, and mitochondria) and large molecule disorders (including lysosomal storage disorders, peroxisomal disorders, glycosylation disorders, and leukodystrophies). Details including key clinical features, salient electrophysiological and neuroradiological findings, biochemical findings, and treatment options are summarized for prominent disorders in each category.

## 1. Introduction

Inherited metabolic epilepsies are disorders that, while individually rare, are in aggregate a substantial clinical portion of child neurology, as well as a complex field of knowledge for physicians, investigators, and students to tackle. A subset of these disorders can lead to the development of epileptic encephalopathy, that is, a brain disturbance due to highly active clinical or electrographic ictal activity. The epileptologist may view these from the viewpoint of syndromic phenotypes such as early myoclonic encephalopathy, early infantile epileptic encephalopathy, infantile spasms, and myoclonic epilepsies. They have various degrees of treatability at present, with some requiring prompt diagnosis and intervention to avoid otherwise catastrophic outcomes. Careful consideration of metabolic disorders in patients presenting with epileptic encephalopathy is therefore warranted, and to this end, we hope a review may be helpful.

This paper provides an overview of inborn metabolic errors associated with epileptic encephalopathy, summarizing key clinical features and underlying biochemistry, salient electrophysiological and neuroradiological findings, and primary treatment options where appropriate. Examples of specific disorders are discussed, with full listings of the multiple enzyme defects and diseases in particular categories presented in tables. The range of inherited metabolic disorders has been organized by the category of molecules or the biochemical process involved: for example, small molecule disorders include dysfunction involving amino, organic, or fatty acids, neurotransmitters, the urea cycle, vitamers and cofactors, and disorders of the mitochondria. Large molecule diseases cover defects in glycosylation, lysosomal and peroxisomal function, and leukodystrophies.

## 2. Small Molecule Disorders

### 2.1. Amino and Organic Acid Disorders

Amino acidopathies and organic acidemias, resulting from disorders in amino or fatty acid catabolism, present with seizures and cognitive, behavioral, or motor disturbances resulting from the accumulation of toxic intermediaries, or possible structural damage [1]. Some may induce an epileptic encephalopathy. Seizure types and EEG findings vary, though myoclonic epilepsies predominate, and diffuse background slowing is a common EEG finding. More typical EEG findings in metabolic encephalopathies are burst suppression, hypsarrhythmia, and generalized spike-wave discharges.  Table 1 lists the protean disorders along with the enzyme defect and metabolites detected on diagnostic studies.

#### 2.1.1. Methylmalonic Acidemia and Cobalamin Deficiencies

**Figure 1:**
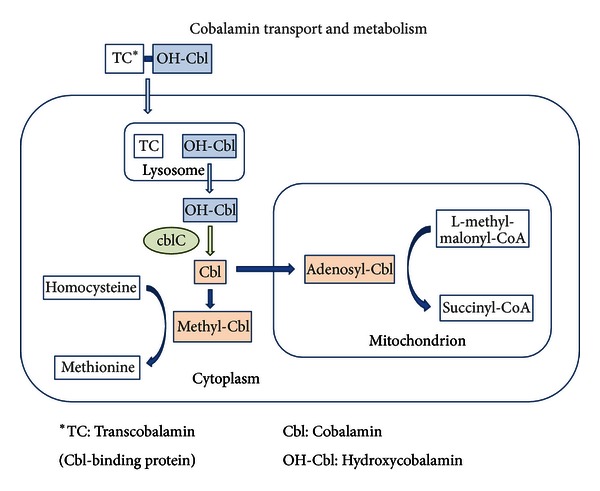
Cobalamin transport and metabolism. Hydroxycobalamin (OH-Cbl) enters the cell bound to transcobalamin (TC), a binding protein. The hydroxycobalamin-transcobalamin complex is broken down inside the lysosome, and the enzyme cobalamin C (CblC) removes the hydroxyl group to generate free cobalamin (Cbl), which is synthesized via additional steps into methyl- and adenosyl-cobalamin.

The finding of elevated methylmalonic acid can be caused by a number of distinct disorders including defects in the vitamin B12-related enzymes cobalamin A, B, C, or D and methylmalonyl CoA mutase (MUT). Early myoclonic encephalopathy, as well as other epilepsies and epileptic encephalopathies, has been associated with this finding. The most common methylmalonic acidemia involving cobalamin is cobalamin C deficiency (Figure 1); individuals may present in infancy or early childhood with seizures and progressive encephalopathy. Patients presenting with status epilepticus have also been reported. Treatment with hydroxycobalamin is effective, including prenatal supplementation in affected families but may not reverse existing neurological injury in delayed diagnoses [3].

#### 2.1.2. Propionic Acidemia

Propionic acidemia (PA) is caused by defects in the enzyme propionyl CoA carboxylase and commonly presents with lethargy, vomiting, metabolic acidemia, and sometimes hyperammonemia [4]. A severe presentation of this disease in infancy with infantile spasms and hypsarrhythmia is reported [5]; myoclonic and generalized seizures are common in early childhood and infancy and typically evolve into mild generalized and absence seizures later in life [6].

#### 2.1.3. Ethylmalonic Acidemia

Ethylmalonic encephalopathy is usually lethal in infancy or early childhood and has a severe presentation including seizures, brain structural malformations, neurodevelopmental regression, pyramidal and extrapyramidal symptoms, chronic diarrhea, and dermatological findings including petechiae and acrocyanosis. MRI has shown frontotemporal atrophy, enlargement of the subarachnoid spaces, and basal ganglia T2-weighted hyperintensities [7]. EEGs may worsen over time, with multifocal spike and slow waves and background disorganization [8].

#### 2.1.4. 3-Hydroxy-3-Methylglutaric Acidemia

When untreated, dysfunction of the enzyme 3-hydroxy-3-methylglutaryl CoA lyase (which cleaves 3-hydroxy-3-methylglutaryl CoA into acetyl CoA and acetoacetate) leads to metabolic acidosis with absent ketone production, lactic acidemia, hypoglycemia hepatomegaly, and lethargy, possibly progressing to coma and death. Seizures are linked in most cases to lactic acidemia or hypoglycemia and are associated with multifocal spike-wave discharges on EEG [8]. Some presentations show particular association with white matter lesions, dysmyelination, and cerebral atrophy on neuroimaging [9, 10].

#### 2.1.5. Glutaric Acidemia

Dysfunction of the enzyme glutaryl CoA dehydrogenase prevents the metabolism of tryptophan, hydroxylysine, and lysine, resulting in increased urine glutaric acid metabolites. This is a cerebral organic acidopathy, with predominantly neurological symptoms featuring macrocephaly, increased subarachnoid spaces, and progressive dystonia and athetosis with striatal injury [11]. Seizures can be a presenting sign and are seen during acute encephalopathic events [12, 13]. EEGs show background slowing with generalized spike-and-wave and mixed multifocal discharges [8, 14]. Therapy using a low-protein diet (especially low lysine and tryptophan), carnitine supplementation, and aggressive emergency management can significantly improve the outcomes. The antiepileptic valproate should be avoided, however, because it is believed to affect acetyl CoA/CoA ratios and may exacerbate metabolic imbalance [13].

#### 2.1.6. 3-Methylglutaconic Acidurias

Five subtypes of 3-methylglutaconic aciduria (MGA) have been categorized (Table 1); all are cerebral organic acidopathies resulting from defects in the leucine catabolic pathway. Neurological and developmental symptoms are central in all five types although seizures are prominent mainly in Type I. Patients with Type I MGA present with leukoencephalopathy and epileptic encephalopathy with psychosis and depression, in addition to ataxia, optic atrophy, and sensorineural hearing loss. These are often accompanied by systemic issues including cardiomyopathy, liver and exocrine pancreatic dysfunction, and bone marrow failure [15]. EEGs show diffuse slowing and white matter lesions can be seen on MRI, usually in a supratentorial location [8, 15, 16].

#### 2.1.7. Canavan Disease

Canavan disease is primarily a disease of demyelination. It is thought to be caused by brain acetate deficiency resulting from a defect of N*-*acetylaspartic acid (NAA) catabolism [17]. Accumulation of NAA, a compound thought to be responsible for maintaining cerebral fluid balance, can lead to cerebral edema and neurological injury. Presentation of Canavan disease includes progressive epileptic encephalopathy with developmental delay, macrocephaly, leukodystrophy, and optic atrophy [18, 19]. Seizures often begin in the second year of life, and treatment is primarily supportive, including antiepileptic medications.

#### 2.1.8. D- and L-2-Hydroxyglutaric Aciduria

D-2-Hydroxyglutaric aciduria results from the loss of enzyme function in either D-2-hydroglutaric dehydrogenase or hydroxyacid-oxoacid transhydrogenase, which metabolizes GHB (gamma-hydroxybutyrate). Though presentations vary, severe cases manifest in the neonatorum with encephalopathy, intractable epilepsy, and cardiomyopathy. Seizures are present in almost all cases, and MRI findings have included signal alterations in the basal ganglia and diencephalon, as well as agenesis of the corpus callosum.

The enantiomer, L-2-Hydroxyglutaric aciduria, is a disorder of alpha ketoglutarate synthesis. It presents with neurodevelopmental delay, generalized seizures, and progressive ataxia. Multifocal spike-waves and burst suppression can be seen on EEGs [20, 21], and characteristic MRI findings include cerebellar atrophy, subcortical white matter loss, and symmetric T2-weighted hyperintensities in the cerebellar dentate nuclei, globus pallidi, and thalami.

#### 2.1.9. Fumaric Aciduria

Seizures are a prominent feature in fumaric aciduria, due to a defect in the conversion of fumarate to malate. The disorder can present prenatally with polyhydramnios and cerebral ventriculomegaly and manifests in infancy and early childhood with epilepsy, serious neurodevelopmental delay, macrocephaly, opisthotonus, and vision loss. Status epilepticus is well reported. Diffuse polymicrogyria, decreased white matter, large ventricles, and open opercula are seen on neuroimaging [22].

#### 2.1.10. Maple Syrup Urine Disease and Dihydrolipoamide Dehydrogenase Deficiency

**Figure 2:**
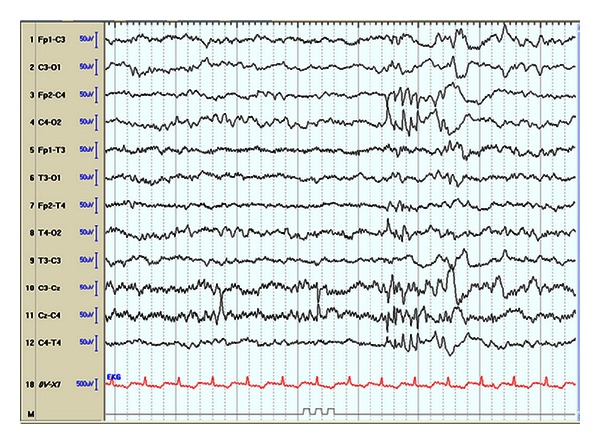
EEG of 4-day-old infant with MSUD shows comb-like rhythm over the right central (C4) area. (sensitivity 7 mcv/mm, HFF 70 Hz, time constant 0.5 sec, 8 sec epoch). Pearl [2].

Dysfunction of the enzyme branched-chain 2-keto dehydrogenase (BCKD) in maple syrup urine disease (MSUD) prevents normal degradation of the branched-chain amino acids leucine, isoleucine, and valine, leading to toxic accumulation of metabolites. Neurological symptoms present in infancy and include cerebral edema, seizures, lethargy, vomiting, and “bicycling” movements [23]. Seizures are related to the cerebral edema and hyperlycinemia, and these symptoms can progress to coma and death. The EEG may show a characteristic comb-like rhythm (Figure 2). Treatment focuses on removing leucine from blood with dialysis or by reversing catabolism through feeding.

Dihydrolipoamide dehydrogenase deficiency, sometimes referred to as MSUD Type III, is due to a defect in a subunit of BCKD, as well as 3 other essential enzymes. The disorder leads to metabolic acidosis and neurological injury, and patients can present with hypoglycemia, absent ketones, elevated liver transaminases, and seizures [24]. The disorder is often fatal at an early age, representing multienzyme failure.

### 2.2. Disorders of GABA Metabolism

Seizures are an important problem in disorders of the synthesis or degradation of gamma-aminobutyric acid (GABA), the brain's primary inhibitory neurotransmitter. The most common of these is succinic semialdehyde dehydrogenase (SSADH) deficiency, though GABA-transaminase deficiency—while extremely rare—features a more severe, progressive epileptic encephalopathy. Both are inherited metabolic disorders affecting GABA degradation.

#### 2.2.1. Succinic Semialdehyde Dehydrogenase Deficiency (4-Hydroxybutyric Aciduria)

**Figure 3:**
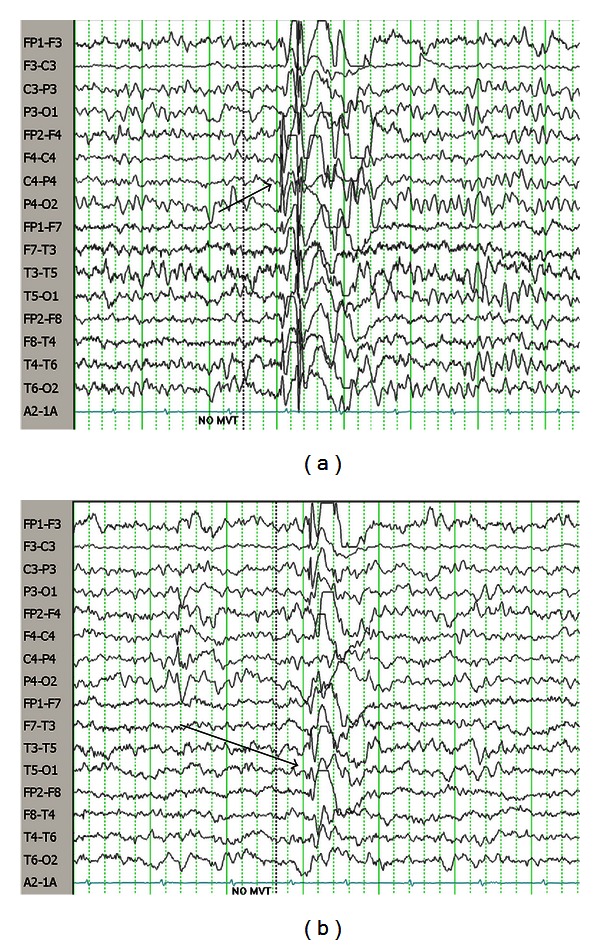
(a) EEG of 3-year-old female with SSADH deficiency. Note diffuse spike-wave paroxysm with lead-in over right hemisphere. (b) Same recording as top, showing left-sided spike-wave paroxysm. Pearl [2].

Deficiency of the enzyme succinic semialdehyde dehydrogenase (SSADH) results in increased systemic and CSF levels of GABA and its catabolite, 4-hydroxybutyric acid (GHB). Patients present with neurodevelopmental delay, expressive language impairment, hypotonia, hyporeflexia, ataxia, and behavioral disorders that commonly include obsessive compulsive and attention deficit hyperactivity disorders. Over 50% of individuals with SSADH deficiency will develop seizures, most commonly tonic-clonic and atypical absence seizures [25]. Recurring status epilepticus and sudden unexpected death in epilepsy patients (SUDEP) have been reported, the latter associated with escalating seizure frequency and severity [26]. MRIs show increased T2-weighted signals in the globus pallidus, cerebellar dentate nucleus and subthalamic nucleus, and variably cerebral and cerebellar atrophy [27]. EEGs typically show generalized spike-wave activity although some may have partial features and variable hemispheric lateralization (Figure 3). Treatment is currently limited to antiepileptic and behavioral medications. While valproate is generally avoided due to its ability to inhibit any residual enzymatic activity, its use has been associated with improvement in some patients with challenging epilepsy including epileptic encephalopathy.

### 2.3. Fatty Acid Oxidation Disorders

Severe seizures can be a presenting sign of defects in fatty acid beta-oxidation, a biochemical process that produces alternatives source of acetyl-CoA and ketone bodies for energy. Fatty acid oxidation (FAO) disorders (Table 2) are a large category of diseases that particularly endanger the CNS and other organ systems that have high energy demands. Metabolic decompensation can be triggered by physiological stressors such as fasting, fever, or physical exertion, and symptoms can appear at any age. Acute crises resemble Reye syndrome, with cardiomyopathy and arrhythmia, as well as rhabdomyolysis and hypoketotic hypoglycemia [28, 29].

Blood and urine laboratory tests are informative towards a diagnosis, particularly if done while the patient is symptomatic. Treatment is dependent on clinical presentation and include avoidance of fasting with frequent low-fat and carbohydrate-rich intake for nonsymptomatic patients and moderation of physical stress in combination with medium-chain triglyceride (MCT) supplementation for patients symptomatic with myopathy. Patients in metabolic crisis require close management in an inpatient setting, with immediate reversal of the catabolism [30, 31].

### 2.4. Mitochondrial Diseases

Epilepsy is a common secondary feature of mitochondrial disease, and disorders in this category (Table 3) have been associated with severe epileptic encephalopathy. Seizures can be a logical consequence of mitochondrial dysfunction: deficient energy generation disrupts the active maintenance of neuronal membrane potential, and seizure-induced cellular hyperactivity adds further oxidative stress to already deficient ATP generation. Up to 60% of patients with mitochondrial disease develop seizures, and many of these can be refractory to treatment [32]. Myoclonic epilepsies are the most commonly reported, but almost all seizure types have been seen, and individual patients can often show multiple types.

#### 2.4.1. POLG1 Disease

**Figure 4:**
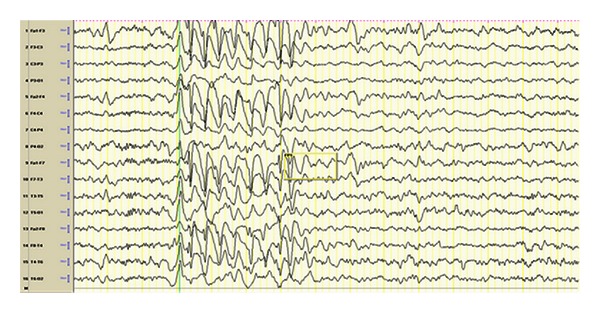
7-year-old boy diagnosed with Alpers' syndrome presented with encephalopathy, tonic-clonic seizures and myoclonic seizures. EEG shows high amplitude anterior poorly formed 2-3 Hertz sharp and slow activity. Pearl [2].

Mutations in the gene *POLG1, *which facilitates mitochondrial DNA replication, have been linked to a range of disease phenotypes, the most prominent being Alpers' disease [33] (Figure 4). Alpers-Huttenlocher disease is a rapidly progressive encephalopathy, causing intractable epilepsy and diffuse neuronal degeneration. Partial complex and myoclonic seizures are most common, although the disorder can evolve to include multiple seizure types [34]. The use of valproate is contraindicated; it has been associated with liver failure in patients with POLG1 mutations, as well as epilepsia partialis continua.

A syndrome of myoclonic epilepsy myopathy sensory ataxia (MEMSA) has also been seen in patients with mutations in POLG1. Seizures, usually focal and often refractory, begin in young adulthood, though other symptoms including sensory neuropathy and cerebellar ataxia may occur in adolescence. Over time, individuals develop cognitive decline and myopathy [35].

#### 2.4.2. Myoclonic Epilepsy with Ragged Red Fibers (MERRF)

Myoclonic epilepsy with ragged red fibers (referring to the appearance of affected muscle cells) is a progressive epilepsy syndrome associated with prominent myoclonus, cognitive decline, optic atrophy, hearing loss, and myopathy [36]. It is associated with mtDNA mutations, and symptoms usually become noticeable in adolescence or young adulthood [37]. EEG findings include focal discharges, atypical spike- or sharp- and slow-wave discharges, and suppression of this activity during sleep [38].

#### 2.4.3. Leigh Syndrome

Leigh syndrome, also known as subacute necrotizing encephalomyopathy, can be seen in disorders involving mitochondrial DNA (as well as nonmitochondrial disorders). Individuals present with neurologic regression with worsening hypotonia, spasticity, and brainstem failure as the disease progresses. Neuroimaging may show bilateral, symmetric lesions in the basal ganglia, thalami, midbrain, and brainstem as well as cortical and cerebellar atrophy [39]. Focal and generalized epilepsy are associated with this phenotype, and epilepsia partialis continua, as well as infantile spasms and hypsarrhythmia, has been described [40].

### 2.5. Cerebral Folate Deficiency

Cerebral folate deficiency can be a common end result of diverse metabolic and genetic conditions (Table 4). A suspected pathology in primary CFD involves impaired transport of folate across the choroid plexus into the central nervous system. This may be due to one of multiple causes, including loss of function mutations in the folate FR1 receptor, blocking autoantibodies to the folate receptor, or disrupted uptake due to valproic acid. Secondary folate deficiency can also be seen in inborn metabolic diseases, such as Rett syndrome, 3-phosphoglycerate dehydrogenase deficiency (a congenital serine biosynthesis disorder), and mitochondrial disorders such as Kearns-Sayre or Alpers disease [41–44].

Primary cerebral folate deficiency (CFD) is characterized by normal blood but low CSF levels of 5-methylhydrofolate (5-MTHF), the physiologically active form of folate. The common phenotype includes epilepsy, along with neurodevelopmental delay (or regression) and dyskinesias. Individuals with blocking autoantibodies to folate receptors present in early childhood with intractable generalized tonic-clonic seizures. In some cases, treatment with high doses of folinic acid (as opposed to folic acid, which has poor blood-brain barrier entry) has been reported to ameliorate seizures and improve neurological function [45].

### 2.6. Serine Synthesis Defects

Lserine, a nonessential amino acid, is synthesized from 3-phosphoglycerate by the sequential activity of three enzymes, each with associated disorders (Table 5). The majority of patients with serine deficiencies are affected by an abnormality in the first step, 3-phosphoglycerate dehydrogenase. The clinical phenotype, which includes congenital microcephaly and psychomotor retardation with refractory seizures and hypsarrhythmia, is nonspecific, likely leading practitioners to suspect in utero processes such as TORCH (Toxoplasmosis, Syphilis, Rubella, Cytomegalovirus, Herpes simplex, HIV) or static perinatal difficulties. MRI findings in infantile onset patients have revealed cortical and subcortical atrophy, as well as delayed myelination [46]. Juvenile onset has also been reported, with presentation at school age with absence seizures and moderate developmental delay [47]. Supplementation with oral serine and glycine has been reported to significantly improve seizures, spasticity, behavior, and feeding, as well as white matter volume and myelination [48, 49].

### 2.7. DEND Syndrome (Developmental Delay, Epilepsy, and Neonatal Diabetes)

A syndrome that combines the problems of developmental delay, epilepsy, and neonatal diabetes is an epileptic channelopathy associated with mutations in potassium channel and sulfonylurea receptor genes [50]. These mutations permanently “lock in” the K_ATP_ channel in an open state, leading to insufficient insulin release and severe hyperglycemia within the first six months of life [51–53]. Clinical manifestations include neurodevelopmental delay, dysmorphic features, hypotonia, and seizures starting as early as the neonatorum. Infantile spasms with hypsarrhythmia, as well as severe tonic-clonic and myoclonic epilepsies, are reported. Neonatal hyperglycemia in DEND can be managed with insulin or sulfonylureas, but the latter is capable of bypassing the defective regulation of K_ATP_ channels and may have better efficacy for the neurological phenotype [50, 54, 55].

### 2.8. Hyperinsulinism-Hyperammonemia (HI-HA)

HI-HA is a syndrome of congenital hyperinsulinism and hyperammonemia that has been related to activating mutations affecting GDH (glutamate dehydrogenase), a participant in the insulin secretion pathway. These defects cause GDH to become insensitive to inhibition, resulting in excess ammonia production and insulin release and neurological sequelae from hypoglycemic insults and hyperammonemia [56]. The clinical constellation of generalized epilepsy, learning disorders, and behavior problems, in the context of hypoglycemia (both postprandial and fasting) and persistent hyperammonemia, is characteristic.

Hypoglycemic seizures may be the first apparent indication. However, some patients have been observed to experience paroxysms, accompanied by generalized electroencephalographic features, without hypoglycemic episodes. This suggests that epilepsy in HI-HA may not be due solely to low CNS glucose availability [57–59]. HI-HA is manageable with a combination of dietary protein restriction, glucagon, antiepileptic medications, and diazoxide, a K_ATP_ channel agonist that inhibits insulin release [60].

### 2.9. Glucose Transporter 1 Deficiency

Glucose transporter type I (Glut-1) facilitates the passage of glucose across the blood-brain barrier, and its dysfunction in the developing brain leads to the development of a metabolic encephalopathy. CSF shows hypoglycorrhachia associated with normal plasma glucose and low-to-normal CSF lactate, measured in a fasting state. A wide array of phenotypes has been associated with this disorder, but 90% of affected children develop epilepsy (of various types, including absence, focal, generalized myoclonic, clonic, tonic, and nonconvulsive status epilepticus) [61]. Microcephaly, ataxia, and psychomotor delay may be present [62], but patients may also suffer from epilepsy without any accompanying motor or cognitive deficiencies. Haploinsufficiency (of the SLC2A1 gene) is correlated with the severity of symptoms [63]. EEG findings vary and may be normal, but usually include either focal or generalized slowing or attenuation, or spike-and-wave discharges (generalized, focal, or multifocal). Neuroimaging results may demonstrate diffuse atrophy.

Glucose transporter I deficiency has emerged as the leading metabolic indication for the ketogenic diet, a dietary therapy that replaces glucose with ketone bodies as the primary biochemical energy source. Response is rapid, even in the case of formerly refractory seizures, and treatment should be maintained long term. Additionally, there are certain compounds known to inhibit Glut-1, including phenobarbital, diazepam, methylxanthines (theophylline, caffeine), and alcohol, which should be avoided [64].

### 2.10. Pyridoxine, Folinic Acid, and Pyridoxal-5′-Phosphate Dependent Epilepsies

There are various epileptic encephalopathies related to vitamin B6 metabolism (Table 6), and pyridoxine-dependent epilepsy (PDE) is the prototype, resulting from a loss of the biologically active pyridoxal-5′-phosphate (PLP) due to a dysfunction of the protein antiquitin (*ALDH7A1*) [65]. PDE normally presents within the first hours following birth with serial refractory seizures responsive to pyridoxine administration. Improvement is significant and usually rapidly appreciable on EEG [66]. Variants of the disorder that respond to folinic acid instead of, or in addition to, pyridoxine have also been described, as well as atypical cases with long asymptomatic periods or presenting later in infancy (i.e., weeks or months following birth) [67, 68].

PNPO, or pyridox(am)ine phosphate oxidase, deficiency is a distinct disorder involving refractory seizures responsive not to pyridoxine but to its biologically active form, pyridoxal-5′-phosphate (PLP) [69, 70]. This disorder is due to a defect in the enzyme PNPO, which synthesizes PLP from precursors pyridoxine-P and pyridoxamine-P [71]. Patients may present prenatally with fetal seizures and premature birth and if untreated can progress to status epilepticus and death. Laboratory and genetic testings are available to confirm these diagnoses (Table 6); trials of systemic pyridoxine administration require close cardiorespiratory monitoring.

### 2.11. Urea Cycle Disorders

The urea cycle (Figure 6), the metabolic mechanism for nitrogen detoxification and removal, is facilitated by six enzymes and a mitochondrial transporter and carrier, each being susceptible to dysfunction (Table 7). In the event of an enzyme or transport defect, the resulting hyperammonemia can lead to overwhelming encephalopathy, often accompanied by seizures and hypotonia that may be exacerbated by metabolic stresses such as fever or infection [72]. EEG monitoring should be initiated early in the course of acute treatment, as seizure activity is thought to be related to hyperammonemic crises or structural damage, and subclinical electrographic seizures are reported. Males with OTC deficiency typically present in the neonatorum and with high mortality, whereas female heterozygotes can vary in the severity and timing of presentation depending on hepatic lyonization [72].

The goals of therapy during metabolic crisis are removal of ammonia through hemodialysis, nitrogen scavenging with agents including sodium benzoate and sodium phenylacetate [73], and reversal of catabolism. Immediate antiepileptic therapy is indicated for optimizing treatment; valproate, however, may interfere with the urea cycle and precipitate metabolic crises [74]. Maintenance therapy of urea cycle defects hinges on the restriction of protein intake while providing sufficient essential amino acids. Orthotopic liver transplant may be curative, but cannot reverse existing neurologic injury.

### 2.12. Creatine Biosynthesis and Transport Deficiencies

Half of the body's daily requirement of creatine is synthesized from arginine and glycine by the enzymes AGAT (arginine:glycine amidinotransferase) and GAMT (guanidino acetate methyl transferase). A specific creatine transporter, CT1, encoded by an X-linked gene, facilitates the uptake into tissues. Patients with deficiencies in creatine synthesis or transport present with early developmental delay, seizures, neurologic regression, intellectual disability, autistic behavior, hypotonia, and movement disorders. Females with heterozygous mutations of the creatine transporter gene may be symptomatic with more moderate intellectual disability, learning and behavior problems, and epilepsy [75].

Approximately half of individuals with GAMT deficiency, and most males with creatine transporter deficiency, develop epilepsy [76]. Patients with GAMT deficiency have abnormal MRI signals of the globus pallidus and background slowing and generalized spike-and-wave discharges on EEG. Individuals with creatine transporter deficiency present with generalized and partial epilepsy, with EEG usually reported as showing generalized polyspike or multifocal epileptiform discharges [77, 78].

Laboratory identification using urine creatine metabolites (Table 8) is necessary to distinguish the three disorders [79]. In the case of creatine synthesis disorders, treatment with oral creatine supplementation can improve seizures and neurological function, and arginine restriction and ornithine supplementation are utilized in GAMT deficiency. Creatine transport disorders, however, are not significantly amenable to therapy other than with traditional antiepileptic medications.

### 2.13. Glycine Encephalopathy

Glycine encephalopathy is an inherited disorder of glycine degradation resulting from defects in the mitochondrial glycine cleavage system (GCS). The excitatory effects of glycine on the cortex and forebrain, mediated by *N-*methyl-D-aspartate(NMDA) receptors, lead to excess intracellular calcium accumulation and subsequent neuronal injury with intractable seizures [80]. Based on age at presentation and clinical outcomes, different categories of glycine encephalopathy (GE) can be distinguished. The majority of patients present with a severe neonatal-onset form, with primarily myoclonic and intractable seizures, hypotonia, apnea, and coma [81]. Outcomes are generally poor, particularly in the presence of brain malformations such as corpus callosum hypoplasia. There are attenuated forms, lacking congenital malformations, with a better outcome.

**Figure 5:**
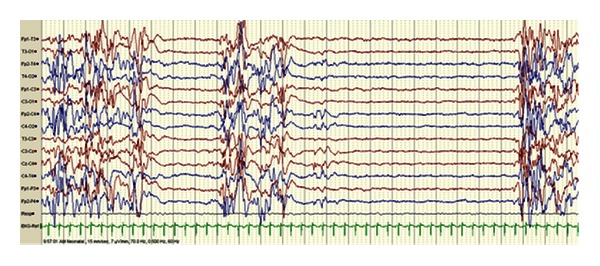
EEG of term infant with glycine encephalopathy shows burst-suppression pattern. Pearl [2].

**Figure 6:**
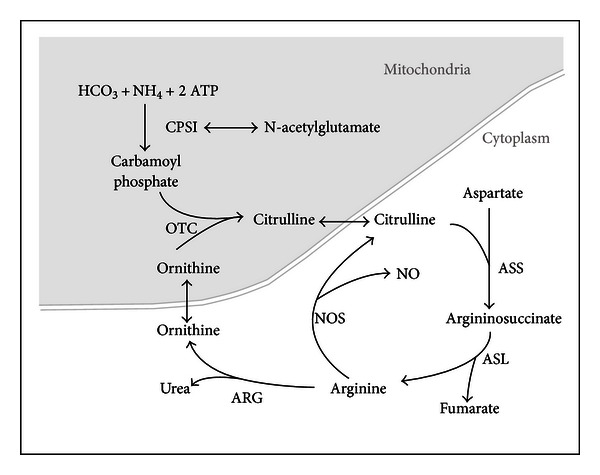
The urea cycle. The urea cycle is a pathway of cytosolic and mitochondrial proteins involved in the conversion of ammonia (NH_4_) to urea for excretion via the kidneys. The enzymes in the pathway are as follows: carbamoylphosphate synthetase (CPS1), ornithine transcarbamylase (OTC); argininosuccinic acid synthase (ASS); argininosuccinic acid lyase (ASL); arginase I (ARG); nitric oxide synthase (NOS). Pearl [2].

Laboratory analyses reveal elevated plasma and CSF glycine, as well as an increased CSF to plasma glycine ratio. EEG findings include multifocal epileptiform activity, hypsarrhythmia, and burst-suppression patterns (Figure 5) [82]. Treatment with benzoate and a low-protein diet may reduce glycine levels in plasma, and combined antiepileptic treatment is necessary for most individuals.

### 2.14. Sulfide Oxidase Deficiency/Molybdenum Cofactor Deficiency

Sulfite oxidase deficiency due to molybdenum cofactor deficiency (MOCOD) and isolated sulfite oxidase deficiency (ISOD) are inherited disorders of the metabolism of sulfated amino acids [83]. They typically present in the first days of life with poor feeding following an uneventful pregnancy and delivery. Seizures, primarily myoclonic or tonic-clonic, begin in the first few weeks of life; they can be refractory to therapy and may develop into status epilepticus [84]. Signs of encephalopathy with opisthotonus, apnea, prolonged crying, and provoked erratic eye movements or myoclonias can be seen, and up to 75% of patients will have slight dysmorphia including widely spaced eyes, small nose, puffy cheeks, and elongated face [85].

EEG findings include burst suppression patterns and multifocal spike-wave discharges [86], and neuroimaging results are usually profoundly abnormal, including diffuse cerebral edema evolving into cystic lesions and brain atrophy within weeks [87]. Low total plasma homocysteinemia is associated with both ISOD and MOCOD, and hypouricemia due to secondary xanthine dehydrogenase deficiency can be indicative of MOCOD. Therapy has historically been symptomatic, with combination or monoantiepileptics.

### 2.15. Homocysteinemias

Disorders involving homocysteine metabolism, specifically methionine and cystathionine synthesis, are characterized by elevated urine and serum homocysteine in the context of neurological symptoms. The spectrum of neurological dysfunction in homocysteine metabolism disorders is wide, including epilepsy, encephalopathy, peripheral neuropathy, ataxia, microcephaly, and psychiatric disorders. Cystathionine beta-synthetase (CBS) deficiency is the most common of the homocysteinemias, with severe defects involving multiple systems due to the essentiality of homocysteine and methionine to normal biochemical function. Focal seizures, stroke, neurodevelopmental delay, and cognitive deficiency, as well as psychosis are common neurological findings. Marfan-like skeletal symptoms, connective tissue abnormalities in the optic lens, and vasculopathies causing thrombosis and multiorgan infarcts may also be present. Treatment and biochemical findings in CBS, as well as other homocysteine disorders are summarized in Table 9.

Autosomal recessively inherited deficiency of methylene tetrahydrofolate reductase (MTHFR) may present in early infancy with severe epileptic encephalopathy [88]. The presentation with hypotonia, lethargy, feeding difficulties, and recurrent apnea may progress from seizures to coma and death. Infantile spasms may also be the presenting feature, with evolution to multiple seizure types including the Lennox-Gastaut syndrome. Status epilepticus, both clinical and subclinical, has been reported. Progressive microcephaly and global encephalopathy may ensue as seizures continue, but there is evidence for reversibility with treatment comprised principally of the methyl donor betaine [89].

### 2.16. Purine and Pyrimidine Defects

Disorders of purine and pyrimidine metabolism may present with epileptic encephalopathies (Table 10), including adenylosuccinase (adenylosuccinate lyase) deficiency which has a broad phenotypic spectrum including neonatal seizures [90]. Lesch-Nyhan disease, or X-linked hypoxanthine-guanine phosphoribosyltransferase deficiency, may result in epileptic seizures, but these can be difficult to distinguish from the extrapyramidal manifestations, specifically dystonic spasms, tremor, and myoclonus. Generalized tonic-clonic seizures are the most commonly reported epilepsy type in the literature [91, 92]. Treatment with allopurinol is essential for hyperuricemia and may provide some antiepileptic effect. Antiepileptic drug choices must weigh the possibility of exacerbating underlying behavioral irritability with levetiracetam and others. Topiramate and zonisamide are avoided due to the risk of nephrolithiasis.

## 3. Large Molecule Disorders

### 3.1. Disorders of Glycosylation

Disorders of protein glycosylation, due to defects in the synthesis of N- and O-linked glycoproteins, are characterized by multiple organ system dysfunction, developmental delay, hypotonia, and epilepsy. Certain of these disorders are associated with severe encephalopathy, particularly those involving alpha-dystroglycan, a protein component of the extracellular matrix, essential to muscle integrity. These are known as dystroglycanopathies.

#### 3.1.1. Walker-Warburg Syndrome

Walker-Warburg syndrome (WWS) is a severe dystroglycanopathy which can present at birth or prenatally with hydrocephalus and encephaloceles on imaging. Seizures and significant structural abnormalities (e.g. cerebellar atrophy, hypoplasia of the corpus callosum), migrational defects (type II lissencephaly), hypomyelination, and ophthalmologic defects are seen. Life expectancy is less than three years [93, 94].

#### 3.1.2. Fukuyama Congenital Muscular Dystrophy

Fukuyama congenital muscular dystrophy (FCMD) classically presents in the neonatorum or even prenatally (poor fetal movement) with frequent seizures and severe brain abnormalities (migration defects, cobblestone lissencephaly, delayed myelination, hypoplasia of the pons, cerebellar cysts). By the age of 3, most patients develop epilepsy. Muscular degeneration and cardiac involvement are progressive [95, 96].

### 3.2. Lysosomal Storage Disorders

Lysosomal storage disorders (LSD) are a major category of diseases that involve defects in lysosomal enzyme function, lysosomal biogenesis, activation, trafficking, or membrane transporters. Over two-thirds of LSDs are neurodegenerative and some are associated with epileptic encephalopathy.  Table 11 lists lysosomal storage disorders by subgroup, and prominent examples are covered below.

#### 3.2.1. Neuronal Ceroid Lipofuscinoses (NCLs)

Neuronal ceroid lipofuscinoses are genetically heterogeneous neurodegenerative disorders associated with defects in transmembrane proteins and are characterized by the accumulation of autofluorescent lipopigments in lysosomes. The symptoms include cognitive decline, seizures, vision loss, and motor impairment, though age of onset and clinical course vary [97]. The development of epilepsy can indicate a more severe clinical course, though myoclonic seizures should be distinguished from myoclonus, which is also a frequent and sometimes progressive feature of this disorder [98]. Generalized cerebral and cerebellar atrophy can be seen on neuroimaging [99].

#### 3.2.2. Sphingolipidosis and Gaucher Disease

Defects in the degradation of sphingolipids, an essential component of myelin sheaths and neuronal tissue, lead to progressive neurodegeneration, epilepsy, peripheral neuropathy, extrapyramidal symptoms, and characteristic “cherry-red spots.” Subtypes (II and III) of Gaucher Disease, which result from a deficiency in glucocerebrosidase, can cause devastating and rapid neurological deterioration. Neuroimaging is usually normal in patients with Gaucher Disease, but EEG can show several abnormalities including polyspikes with occipital predominance sensitive to photostimulation, diffuse slowing with high-voltage sharp waves during sleep, and multifocal spike-and-wave paroxysms [100, 101].

#### 3.2.3. Gangliosidosis and Tay-Sachs Disease

Tay-Sachs disease is an example of defects involving the degradation of gangliosides, which are vital signaling, transport, and regulatory proteins in the lysosomal membrane. Seizures typically begin within the first year of life and worsen in frequency and severity. They are difficult to control with antiepileptics and can acutely and rapidly progress; in these cases, EEG and clinical deterioration can follow until death [102].

### 3.3. Peroxisomal Diseases

As a participant in cellular detoxification, lipid metabolism, as well as myelin, neuronal function, migration, and brain development [103], peroxisomes are essential for neuronal health. Nearly all peroxisomal disorders (Table 12) are known to impair neurological function, though peroxisomes are present in almost all eukaryotic cells, and consequently its associated diseases will also manifest in multiple organ systems. Seizures occur particularly in the neonatal period and may be a result of cortical migration defects. Symptomatic treatment using anticonvulsants is the predominant therapy [104, 105].

#### 3.3.1. Rhizomelic Chondrodysplasia Punctata

Rhizomelic chondrodysplasia punctata is a peroxisomal biogenesis disorder characterized by white matter abnormalities including inflammatory demyelination and noninflammatory dysmyelination, as well as cerebellar degeneration and loss of Purkinje cells, leading to profound intellectual deficiency. Almost all patients develop seizures, with nonspecific EEG findings. Neurological defects and degeneration are severe, and most individuals do not survive the first two years of life [106].

### 3.4. Leukodystrophies

Genetic leukoencephalopathies, or inherited white matter disorders, are diseases that primarily affect myelinated structures in the brain and peripheral nervous system. The majority of leukodystrophies primarily feature motor dysfunction rather than encephalopathy, particularly early on in the development of the disease. Epilepsy, however, can be a prominent symptom in certain classic leukodystrophies (Table 13) such as Alexander's disease. This is associated with defects in the GFAPgene encoding astrocyte intermediate filaments. Early onset forms of Alexander's disease (Type I) frequently feature seizures, particularly associated with fever, that are difficult to control. The clinical course of type I Alexander's disease is normally progressive neurodegeneration involving megalencephaly, psychomotor retardation, and spastic paraplegia [107].

## 4. Conclusion

Epileptic encephalopathies represent a challenging area of pediatric neurology and epilepsy and have a broad differential diagnosis [108]. There are protean inborn errors of metabolism which may lead to epileptic encephalopathies. They have various degrees of treatability at present, with some requiring prompt diagnosis and intervention to avoid otherwise catastrophic outcomes. The epileptologist may view these from the viewpoint of syndromic phenotypes. In general, early myoclonic encephalopathy and myoclonic seizures represent a classic epilepsy syndrome and seizure type, respectively, associated with inborn errors of metabolism. Yet, the phenotypic spectrum of epilepsy caused by hereditary metabolic disorders is wide and includes refractory neonatal seizures, early infantile epileptic encephalopathy (syndrome of Ohtahara), infantile spasms, and progressive myoclonic epilepsies, as well as syndrome variations such as early onset absence epilepsy in glucose transporter deficiency. A careful approach to metabolic disorders is helpful to consider the various diseases that may present and develop into an epileptic encephalopathy.

The small molecule disorders include amino and organic acidopathies such as maple syrup urine disease, homocysteinemia, multiple organic acid disorders, and cobalamin deficiencies. Dietary intervention is key in preventing encephalopathy in maple syrup urine disease and glutaric aciduria, and hydroxycobalamin has a therapeutic role starting in prenatal intervention in cobalamin C deficiency. Neurotransmitter and fatty acid oxidation disorders may result in epileptic encephalopathies, and mitochondrial disorders present with a range of epilepsy phenotypes, including intractable epilepsy and epilepsia partialis continua in polymerase gamma mutations. Cerebral folate deficiency appears to result from a variety of causes but primary deficiency, associated with mutations of the folate receptor or blocking antibodies, has a phenotype of intractable generalized tonic-clonic seizures in infancy which may respond to folinic acid. Disorders of serine synthesis may respond to pre- and postnatal supplementation with serine and glycine. There are several potassium channelopathies involving the pancreas and brain, presenting either with neonatal diabetes or hypoglycemia, specifically (developmental delay, epilepsy, and neonatal diabetes) DEND and (hyperinsulinism-hyperammonemia) HI-HA with specific therapeutic implications. Glucose transporter deficiency appears to be the prototype of transport defects causing epilepsy and having specific therapy, in this case being the ketogenic diet to supply an alternative brain fuel to glucose, and the disorders related to the pyridoxine vitamers, specifically pyridoxine and pyridoxal-5-phosphate, require prompt identification and therapy to avert a catastrophic outcome. Autosomal recessively inherited deficiency of MTHFR may be reversible with use of betaine, whereas other small molecule defects causing epileptic encephalopathy, for example, glycine encephalopathy and sulfite oxidase deficiency, have no specific therapy at this time.

Large molecule disorders involve a complex constellation of disorders of glycosylation, lysosomal storage diseases, and peroxisomal disorders. Those including severe epilepsy include Walker-Warburg syndrome, Fukuyama congenital muscular dystrophy, gangliosidoses such as Tay-Sachs and Sandhoff diseases, and the neuronal ceroid lipofuscinoses. While epilepsy represents significant gray matter involvement in neurological disease, seizures can be a prominent aspect of leukodystrophies such as Alexander's disease. The epileptologist should consider these hereditary disorders in the investigation of patients with epileptic encephalopathies, leading to specific diagnostic steps and, in some cases, potential therapeutic maneuvers to address the metabolic defect.

## Figures and Tables

**Table 1:** Amino acidemias and organic acidopathies.

Disorder	Defective enzyme	Diagnostic metabolites
Propionic acidemia (PA)	Propionyl CoA carboxylase	Propionylcarnitine (C3; P)* Methylcitrate (U)* 3-Hydroxypropionic acid (U)
Methylmalonic acidemia (MMA)	Methylmalonic mutase Cobalamin A Cobalamin B	Methylmalonic acid (P, U) Propionylcarnitine (C3; P) Methylcitrate (U) 3-Hydroxypropionic acid
Methylmalonic acidemia with homocysteinuria, cobalamin C/D	Cobalamin C Cobalamin D	Methylmalonic acid (P, U) Propionylcarnitine (C3; P) Methylcitrate (U) Total homocysteine (P) 3-Hydroxypropionic acid (U)
Isovaleric acidemia (IVA)	Isovaleryl dehydrogenase	Isovaleric acid (U) Isovalerylcarnitine (C5; P)
3-Methylcrotonylglycinuria (3MCC)	3-Methylcrontonyl CoA carboxylase	3-Hydroxyisovaleric acid (U) 3-Methylcrotonylglycine (U) Hydroxyisovalerylcarnitine (C5OH; P)
3-Hydroxy-3-methylglutaryl CoA lyase deficiency	3-Hydroxy-3-methyl- glutaryl CoA Lyase	Hydroxyisovalerylcarnitine (C5OH; P) 3-Hydroxy-3-methylglutaric acid (U) 3-Methylglutaconic acid (U)
Malonic aciduria	Malonyl CoA decarboxylase	Malonate (U)
2-Methyl-3-hydroxybutyrl CoA dehydrogenase deficiency	2-Methyl-3-hydroxybutyryl CoA dehydrogenase	2-Methyl-3-hydroxybutyrate (U) Tiglylglycine (U)
Ethylmalonic encephalopathy	Branched chain Keto-dehydrogenase	C4 C5 Ethylmalonic acid (U) Methylsuccinic acid C4–C6 acylglycines (P)
Beta-ketothiolase deficiency	3-Methyl acetoacetate thiolase	C5:1 (P) 2-Methyl-3-hydroxybutyrate (U) Tiglylglycine (U) 2-Methyacetoacetate (U)
Biotinidase deficiency and Holocarboxylase synthetase deficiency	Biotinidase Holocarboxylase synthetase	Propionylcarnitine (C3; P) Hydroxyisovalerylcarnitine (C5OH; P) Biotinidase enzyme deficiency (P) Lactate (P, U) 3-Methylcrotonylglycine (U) Methylcitrate (U) 3-Hydroxypropionic acid (U)
2-Methyl butyryl CoA dehydrogenase	2-Methyl butyryl CoA dehydrogenase	2-Methylglycience (U) Isovalerylcarnitine (C5; P)
Glutaric acidemia I	Glutaryl CoA dehydrogenase	Glutaric acid (U) 3-Hydroxyglutaric acid (U) Glutaryl carnitine (C5-DC; P)
3-Methylglutaconic acidurias	3-Methylglutaconyl CoA hydratase (Type I) Barth (Type II) Costeff (Type III) Type IV Type V	3-Methylglutaconic acid (U) Hydroxy-isovalerylcarnitine (P)
Canavan disease	Aspartoacylase	N-Acetylaspartic Acid (U)
L-2-Hydroxyglutaric aciduria	L-2-Hydroxyglutarate dehydrogenase	L-2-Hydroxyglutaric Acid (U) Lysine (CSF)
D-2-Hydroxyglutaric aciduria	D-2-Hydroxyglutarate dehydrogenase Hydroxy-oxoacid transhydrogenase	D-2-Hydroxyglutaric acid (U)
4-Hydroxybutyric Aciduria	Succinate semialdehyde dehydrogenase	Gamma-hydroxybutric acid (U)
Fumaric aciduria	Fumarate hydratase	Fumarate (U)
Maple syrup urine disease (MSUD)	Branched chain Keto-dehydrogenase	Leucine (P) Alloisoleucine (P) Dicarboxylic acids (U)
Dihydrolipoamide dehydrogenase	MSUD III	Leucine (P) Alloisoleucine (P) Dicarboxylic acids (U) Lactic acid (P, U)
Phenylketonuria (PKU)	Phenylalanine hydratase (PAH)	Phenylalanine (P) Low tyrosine (P)

(P): plasma, (U): urine. Adapted from Pearl [2].

**Table 2:** Fatty acid oxidation disorders and biochemical characteristics.

Disorder	Biochemical characteristics
Carnitine uptake defect (CUD) (Primary/systemic carnitine deficiency, carnitine transporter OCTN2 deficiency)	↓↓↓ Carnitine (P)
Carnitine palmitoyltransferase I deficiency (CPT 1A)	↑ Ammonia (P) ↑ Liver enzymes (ALT, AST)
Carnitine palmitoyltransferase II deficiency (CPT II) (i) lethal neonatal (ii) infantile (iii) myopathic	↑ C12–C18 acylcarnitines (P)
Carnitine-acylcarnitine translocase deficiency (CACT)	↑ Ammonia (P) ↑ Liver enzymes (ALT, AST) ↑ Creatine kinase (P) ↑ Long chain acylcarnitines (P) ↓ Free carnitine (P)
Mitochondrial trifunction protein deficiency (TFP) (i) Isolated long chain Acyl-CoA Dehydrogenase deficiency (LCHAD)	Hypoketotic hypoglycemia
Very long chain acyl-CoA dehydrogenase deficiency (VLCAD)	Hypoglycemia (ketotic or nonketotic)
Medium chain acyl-CoA dehydrogenase deficiency (MCAD)	Hypoketotic hypoglycemia
Medium chain 3-ketoacyl-CoA thiolase deficiency (MCKAT)	Ketotic lactic aciduria C6–C12 dicarboxylic aciduria
Short chain acyl-CoA dehydrogenase deficiency (SCAD)	↑ Ethylmalonic acid (U)
Medium/short chain acyl-CoA dehydrogenase deficiency (M/SCHAD)	Hyperinsulinemic hypoglycemia ↑ 3-Hydroxybutylcarnitine ↑ 3-Hydroxy butyric acid (U) ↑ 3-Hydroxy glutaric acid (U)
Glutaric acidemia Type II	Hypoglycemia Metabolic acidosis
2,4-Dienoyl-CoA reductase deficiency	2-Trans,4-Cis-decadienoylcarnitine (P, U)
Acyl-CoA dehydrogenase 9 deficiency (ACAD9)	Persistent lactic acidosis

**Table 3:** Mitochondrial disorders and epilepsy.

Category of disorder	Syndrome
Mitochondrial complex deficiencies	(i) Complex I deficiency (ii) Complex II deficiency (iii) Complex III deficiency (iv) Complex IV deficiency (v) Complex V deficiency

Mitochondrial DNA disorders	(i) mtDNA depletion syndromes (a) POLG1 disease (1) Alpers-Huttenlocher disease (2) Childhood onset epilepsia partialis continua (EPC) (3) Myoclonic epilepsy myopathy sensory ataxia (MEMSA) (ii) mtDNA deletion syndromes (a) Kearns-Sayre syndrome (KSS) (b) Chronic progressive external ophthalmoplegia (CPEO) (iii) Myoclonic epilepsy with ragged-red fibers (MERRF) (iv) Myoclonic epilepsy, lactic acidosis, and stroke (MELAS)

Other associated syndromes	Leigh syndrome

**Table 4:** Cerebral folate deficiencies.

	Disorder or mechanism
Primary cerebral folate deficiency	Folate receptor FR1 defect due to autoantibodies
	Folate receptor FR1 defect due to (FOLR1 gene) mutation
Disorders with secondary cerebral folate deficiency	Aicardi-Goutieres syndrome
	Alpers syndrome
	Isolated Rett syndrome
	Kearns-Sayre syndrome
	Mitochondrial complex I encephalomyopathy
	Valproic acid complications

**Table 5:** Serine synthesis defects.

Disorder	Epilepsy and neuroimaging features	Response to treatment with L-serine and glycine
3-Phosphoglycerate dehydrogenase deficiency	Infantile phenotype:	Infantile phenotype:
	(i) intractable seizures	(i) Seizure control or significantly lowered frequency
	(ii) MRI: hypomyelination and delayed myelination	(ii) Increased white matter volume
	Juvenile phenotype:	Juvenile phenotype:
	(i) absence seizures	(i) Seizure control
	(ii) MRI: no abnormalities	(ii) Prevention of neurological abnormalities
Phosphoserine Aminotransferase deficiency	Symptomatic patient:	Symptomatic patient:
	(i) intractable seizures	(i) No clinical response to treatment
	(ii) MRI: generalized atrophy, including cerebellar vermis and pons, white matter abnormalities	
	Presymptomatic patient:	Presymptomatic patient:
	(i) MRI: no abnormalities	(i) Prevention of all neurological abnormalities
Phosphoserine phosphatase deficiency	Single case, details not reported	Not reported

Adapted from Pearl [2].

**Table 6:** Pyridoxine and pyridoxal-5′-phosphate-dependent Epilepsies.

	Pyridoxine- or folinic-acid-dependent epilepsies (PDE)	Pyridoxal-5′-phosphate (PLP-) dependent epilepsy
Deficient enzyme	Antiquitin (ATQ)	Pyridox(am)ine phosphate oxidase (PNPO)
Blood chemistry	Normal, but hypoglycemia and lactic acidosis have been reported	Hypoglycemia and lactic acidosis common
Vanillactic acid (Urine)	Absent	Present
Pipecolic acid (blood, CSF)	↑	Normal
AASA* (blood, urine, CSF)	↑	Normal
Neurotransmitter metabolites (CSF)	(Possible) ↑ 3-Methoxytyrosine	↑ L-DOPA, 3-Methoxytyrosine ↓ Homovanillic acid, 5-Hydroxyindoleacetic acid
Clinical signs	Postnatal refractory seizures, gastrointestinal symptoms, encephalopathy with hyperalertness, sleeplessness	Fetal distress and in utero fetal seizures, postnatal refractory seizures and encephalopathy

*AASA: alpha-aminoadipic semialdehyde. Adapted from Pearl [2].

**Table 7:** Urea cycle defects and biochemical characteristics.

Defective enzyme or component	Citrulline	Arginine	Ammonia	Additional biochemical characteristics
Ornithine transcarbamylase	↓	↓	↑	↑ Glutamine Normal orotic acid
Carbamoylphosphate synthetase I (CPS1)	↓	↓	↑	↑ Glutamine ↑ Orotic acid
N-acetyl glutamate synthase (NAGS)				Reduced CPS1 activity (NAGS is a vital cofactor)
Argininosuccinate synthase (ASS)	↑++ (10–100x normal)	↓	↑	
Argininosuccinate lyase (ASL)	↑	↓	↑	↑ Argininosuccinic acid (unique to ASL deficiency)
Arginase (ARG1)			Normal in absence of metabolic stress	
Ornithine transporter mitochondrial I (ornithine translocase deficiency)			↑	↑ Homocitrulline ↑ Ornithine
Citrin (solute carrier family 5) deficiency	↑			

Adapted from Pearl [2].

**Table 8:** Creatine synthesis defects.

Defective enzyme or component	Urine Creatine	Urine GAA (guanidinoacetate)	Creatine/creatinine ratio	treatment
AGAT (arginine : glycine amidinotransferase)	↓	↓	Normal	(i) Amenable to creatine therapy
GAMT (guanidine acetate methyl transferase)	↓	↑	Normal	(i) Amenable to creatine therapy; (ii) Dietary restriction of arginine, with ornithine supplementation (iii) Antiepileptics may be necessary for seizure control
Creatine transporter	↑	Normal (may be slightly increased in males)	↑	(i) Antiepileptics for seizure control (ii) Creatine supplementation is ineffective

**Table 9:** Biochemical characteristics and treatment of homocysteine metabolism disorders.

Defective enzyme	Homocysteine (U, P)	Additional biochemical characteristics	Treatment
Cystathionine beta-synthase (CBS)	↑	↑ Methionine ↓ Cysteine	Pyridoxine, B12, folate Methionine-restricted diet Cysteine supplementation betaine
Methionine synthase (MTR)	↑	Normal or ↑ Folate Normal or ↑ Cobalamin	High-dose hydroxycobalamin
Methylene tetrahydrofolate reductase (MTHFR)	↑	↓ Methionine	High-dose betaine Methionine supplementation

*(U): urine, (P): plasma.

**Table 10:** Purine and pyrimidine metabolism disorders involving epilepsy.

Disorder	Defective enzyme	Biochemical characteristics	Seizure characteristics
Lesch-Nyhan Disease (LSD)	hypoxanthine-guanine phosphoribosyl transferase	↑ Uric acid	Predominantly generalized tonic-clonic, developing in early childhood
Adenylosuccinase deficiency	Adenylosuccinate lyase	↑ succinylaminoimidazole carboxamide riboside ↑ succinyladenosine	Neonatal seizures Severe infantile epileptic encephalopathy

**Table 11:** Lysosomal storage disorders.

Storage materials	Diseases	Primary defect
Lipids	Niemann Pick C	Intracellular cholesterol transport
Monosaccharides	Free sialic acid storage disease	Lysosomal transport protein sialin
	(i) infantile free sialic acid storage disease (ISSD)	
	(ii) intermediate salla disease	
	(iii) mild form (salla disease)	
Mucolipidoses	Mucolipidosese	
	(i) type II (I cell disease)	N-acetylglucosamine-1-phosphotransferase
	(ii) type III (pseudo Hurler polydystrophy)	N-acetylglucosamine-1-phosphotransferase
	(iii) type IV	Receptor-stimulated cat ion channel (mucolipidin)
Mucopolysaccharidoses (MPS)	MPS	
Dermatan, heparan sulfate	(i) type IH (Hurler)	L-iduronidase
Dermatan, heparan sulfate	(ii) type II (Hunter)	Iduronate-sulfatase
Heparan sulfate	(i) type III A (Sanfilippo type A)	Heparan-N-sulfatase
	(ii) type III B (Sanfilippo type B)	N-acetyl-*α*-glucosaminidase
	(iii) type III C (Sanfilippo type C)	*α*-glucosaminide-acetyl-CoA transferase
	(iv) type III D (Sanfilippo type D)	N-acetylglucosamine-6-sulfatase
Dermatan, heparan, chondroitin sulphate	(i) type VII (Sly)	*β*-Glucuronidase
Multiple enzyme defects	Multiple sulfatase deficiency	Sulfatase-modifying factor-1 (SUMF1)
	Galactosialidosis	*β*-Galactosidase and neuraminidase secondary to defect of protective protein, cathepsin A
Neuronal ceroid lipofuscinosis (NCL)	NCL	
	(i) congenital	Cathepsin D (CTSD)
	(ii) infantile (INCL)	Palmitoyl-protein thioesterase-1 (PPT1)
	(iii) late infantile (LNCL)	Tripeptidyl peptidase 1 (TPP1)
	(iv) juvenile (JNCL)	A transmembrane protein
	(v) adult (ANCL)	Ceroid lipofuscinosis neuronal protein 3 (CNT3)
	(vi) Northern epilepsy (NE)	Ceroid lipofuscinosis neuronal protein 8 (CLN8)
Oligosaccharidoses (glycoproteinoses)		
	Alpha-mannosidosis	*α*-Mannosidase
	Beta-mannosidosis	*β*-Mannosidase
	Fucosidosis	*α*-Fucosidase
	Schindler disease	*α*-N-acetylgalactosaminidase
	Aspartylglucosaminuria (AGU)	Aspartylglucosaminidase
	Sialidosis	
	(i) severe infantile	*α*-Neuraminidase
	(ii) mild infantile (mucolipidosis I)	*α*-Neuraminidase
	(iii) adult	*α*-Neuraminidase
Sphingolipidoses		
Ceramide	Farber disease	Ceramidase
Galactocerebroside	Globoid Cell Leukodystrophy (GLD or Krabbe disease)	*β*-Galactocerebrosidase
	(i) infantile	
	(ii) late infantile	
	(iii) adult	
	(iv) Saposin A deficiency	Sphingolipid activator protein A (SAPA)
Gangliosidoses	GM1 gangliosidoses	*β*-Galactosidase
	(i) infantile	
	(ii) late infantile	
	(iii) adult	
	GM2 gangliosidoses	*β*-Hexosaminidase
	(i) Sandhoff disease	*β*-Hexosaminidase A and B (*α*-subunit)
	(ii) Tay Sachs	*β*-Hexosaminidase A (*β*-subunit)
	(iii) GM2 activator deficiency	*β*-Hexosaminidase activator
Glucocerebroside	Gaucher disease	*β*-Glucocerebrosidase
	(i) type II	
	(ii) type III	
	(iii) Saposin C deficiency	Sphingolipid activator protein C
Sphingomyelin	Niemann-Pick	Sphingomyelinase
	(i) type A	
	(ii) type B	
Sulfatide	Metachromatic leukodystrophy (MLD)	Arylsulfatase A
	(i) late infantile	
	(ii) juvenile	
	(iii) adult	
	(iv) Saposin B deficiency	Sphingolipid activator protein B
Multiple sphingolipids	Prosaposin deficiency (pSap)	Precursor of Sphingolipid activator protein

Adapted from Pearl [2].

**Table 12:** Peroxisomal disorders.

Biogenesis disorders	Single enzyme disorders	Contiguous gene syndrome
Zellweger spectrum disorders (ZSD) (i) Zellweger syndrome (ZS) (ii) Neonatal adrenoleukodystrophy (NALD) (iii) Infantile refsum disease (IRD) Rhizomelic chondrodysplasia punctata (RCDP)	X-linked adrenoleukodystrophy (X-ALD) Acyl-coA oxidase deficiency Bifunctional protein deficiency (D-BP) Alkyl-DHAP synthase deficiency DHAP-alkyl transferase deficiency Adult Refsum disease (classic) Glutaric aciduria type III Acatalasemia Hyperoxaluria type I	Contiguous ABCD1 DXS1357E deletion syndrome (CADDS)

Adapted from Pearl [2].

**Table 13:** Leukodystrophies including epilepsy as a manifestation.

Disorder	
Alexander's disease	
Globoid cell leukodystrophy (Krabbe disease)	
X-linked adrenoleukodystrophy	
Hereditary diffuse leukoencephalopathy with spheroids	
Metachromatic leukodystrophy	

## References

[1] Köker S, Sauer SW, Hoffmann GF, Müller I, Morath MA, Okun JG (2008). Pathogenesis of CNS involvement in disorders of amino and organic acid metabolism. *Journal of Inherited Metabolic Disease*.

[2] Pearl PL (2013). *Inherited Metabolic Epilepsies*.

[3] Biancheri R, Cerone R, Schiaffino MC (2001). Cobalamin (Cbl) C/D deficiency: clinical, neurophysiological and neuroradiologic findings in 14 cases. *Neuropediatrics*.

[4] Fenton W, Gravel R, Rosenblatt D, Scriver C, Beaudert A, Sly W (2001). Disorders of propionate and methylmalonate metabolism. *The Metabolic & Molecular Basis of Inherited Disease*.

[5] Azuar LA, Viǹas JMP, Crespo PS, Perera JAP, Echeverría MTL (2005). Infantile spasms as the first manifestation of propionic acidemia. *Anales de Pediatria*.

[6] Haberlandt E, Canestrini C, Brunner-Krainz M (2009). Epilepsy in patients with propionic acidemia. *Neuropediatrics*.

[7] Zafeiriou DI, Augoustides-Savvopoulou P, Haas D (2007). Ethylmalonic encephalopathy: Clinical and biochemical observations. *Neuropediatrics*.

[8] Stigsby B, Yarworth SM, Rahbeeni Z (1994). Neurophysiologic correlates of organic acidemias: a survey of 107 patients. *Brain and Development*.

[9] Brismar J, Ozand PT (2009). CT and MR of the brain in the diagnosis of organic acidemias. Experiences from 107 patients. *Neuropediatrics*.

[10] Ozand PT, Al Aqeel A, Gascon G, Brismar J, Thomas E, Gleispach H (1991). 3-Hydroxy-3-methylglutaryl-coenzyme A (HMG-CoA) lyase deficiency in Saudi Arabia. *Journal of Inherited Metabolic Disease*.

[11] Neumaier-Probst E, Harting I, Seitz A, Ding C, Kölker S (2004). Neuroradiological findings in glutaric aciduria type I (glutaryl-CoA dehydrogenase deficiency). *Journal of Inherited Metabolic Disease*.

[12] Strauss KA, Puffenberger EG, Robinson DL, Morton DH (2003). Type I glutaric aciduria, part 1: natural history of 77 patients. *American Journal of Medical Genetics C*.

[13] Kolker S, Christensen E, Leonard J (2011). Diagnosis and managament of glutaric aciduria type I-revised recommendations. *Journal of Inherited Metabolic Disease*.

[14] McClelland VM, Bakalinova DB, Hendriksz C, Singh RP (2009). Glutaric aciduria type 1 presenting with epilepsy. *Developmental Medicine and Child Neurology*.

[15] Engelke UFH, Kremer B, Kluijtmans LAJ (2006). NMR spectroscopic studies on the late onset form of 3-methylglutaconic aciduria type I and other defects in leucine metabolism. *NMR in Biomedicine*.

[16] Illsinger S, Lücke T, Zschocke J, Gibson KM, Das AM (2004). 3-Methylglutaconic aciduria type I in a boy with fever-associated seizures. *Pediatric Neurology*.

[17] Arun P, Madhavarao CN, Moffett JR (2010). Metabolic acetate therapy improves phenotype in the tremor rat model of Canavan disease. *Journal of Inherited Metabolic Disease*.

[18] Canavan MM (1931). Schilder's encephalitis periaxialis diffusa. *Archives of Neurology and Psychiatry*.

[19] Segel R, Anikster Y, Zevin S (2011). A safety trial of high dose glyceryl triacetate for Canavan disease. *Molecular Genetics and Metabolism*.

[20] Chen E, Nyhan WL, Jakobs C (1996). L-2-hydroxyglutaric aciduria: neuropathological correlations and first report of severe neurodegenerative disease and neonatal death. *Journal of Inherited Metabolic Disease*.

[21] Barth PG, Wanders RJA, Scholte HR (1998). L-2-hydroxyglutaric aciduria and lactic acidosis. *Journal of Inherited Metabolic Disease*.

[22] Kerrigan J, Aleck K, T TJ (2000). Fumaric aciduria: clinical and imaging features. *Annals of Neurology*.

[23] Chuang DT, Shih V, Scriver C, Beaudet A, Sly W (2001). Maple syrup urine disease (branched chain ketoaciduria). *The Metabolic and Molecular Basis of Inherited Disease*.

[24] Sansaricq C, Pardo S, Balwani M, Grace M, Raymond K (2006). Biochemical and molecular diagnosis of lipoamide dehydrogenase deficiency in a North American Ashkenazi Jewish family. *Journal of Inherited Metabolic Disease*.

[25] Pearl PL, Jakobs C, Gibson KM, Valle D, Beaudet A, Vogelstein B (2007). Disorders of beta- and gamma-amino acids in free and peptide-linked forms. *Online Molecular and Metabolic Bases of Inherited Disease*.

[26] Knerr I, Gibson KM, Murdoch G (2010). Neuropathology in succinic semialdehyde dehydrogenase deficiency. *Pediatric Neurology*.

[27] Pearl PL, Gibson KM, Cortez MA (2009). Succinic semialdehyde dehydrogenase deficiency: lessons from mice and men. *Journal of Inherited Metabolic Disease*.

[28] Rinaldo P, Matern D, Bennett MJ (2002). Fatty acid oxidation disorders. *Annual Review of Physiology*.

[29] Tein I (2002). Role of carnitine and fatty acid oxidation and its defects in infantile epilepsy. *Journal of Child Neurology*.

[30] Spiekerkoetter U, Bastin J, Gillingham M, Morris A, Wijburg F, Wilcken B (2010). Current issues regarding treatment of mitochondrial fatty acid oxidation disorders. *Journal of Inherited Metabolic Disease*.

[31] Morris A, Spiekerkoetter U, Saudubray J, vanden Berghe G, Walter J (2012). Disorders of mitochondrial fatty acid oxidation and related metabolic pathways. *Inborn Metabolic Diseases: Diagnosis and Treatment*.

[32] Khurana DS, Salganicoff L, Melvin JJ (2008). Epilepsy and respiratory chain defects in children with mitochondrial encephalopathies. *Neuropediatrics*.

[33] Cohen BH, Chinnery PF, Copeland WC, Pagon RA, Bird TD, Dolan CR (1993). POLG-related disorders. *Genereviews*.

[34] Wolf NI, Rahman S, Schmitt B (2009). Status epilepticus in children with Alpers’ disease caused by POLG1 mutations: EEG and MRI features. *Epilepsia*.

[35] Finsterer J (2011). Inherited mitochondrial neuropathies. *Journal of the Neurological Sciences*.

[36] DiMauro S, Schon EA (2008). Mitochondrial disorders in the nervous system. *Annual Review of Neuroscience*.

[37] Johns DR, Flier J, Moller D (1995). Mitochondrial DNA and disease. *The New England Journal of Medicine*.

[38] Canafoglia L, Franceschetti S, Antozzi C (2001). Epileptic phenotypes associated with mitochondrial disorders. *Neurology*.

[39] DiMauro S, De Vivo DC (1996). Genetic heterogeneity in Leigh syndrome. *Annals of Neurology*.

[40] Sabbagh SE, Lebre AS, Bahi-Buisson N (2010). Epileptic phenotypes in children with respiratory chain disorders. *Epilepsia*.

[41] Rosenblatt DS, Fenton WA, Scriver CR, Beaudet AL, Sly WS (2001). Inherited disorders of folate and cobalamin transport and metabolism. *The Metabolic and Molecular Bases of Inherited Disease*.

[42] Dougados M, Zittoun J, Laplane D, Castaigne P (1983). Folate metabolism disorder in Kearns-Sayre syndrome. *Annals of Neurology*.

[43] Pineda M, Ormazabal A, López-Gallardo E (2006). Cerebral folate deficiency and leukoencephalopathy caused by a mitochondrial DNA deletion. *Annals of Neurology*.

[44] Hasselmann O, Blau N, Ramaekers VT, Quadros EV, Sequeira JM, Weissert M (2010). Cerebral folate deficiency and CNS inflammatory markers in Alpers disease. *Molecular Genetics and Metabolism*.

[45] Moretti P, Sahoo T, Hyland K (2005). Cerebral folate deficiency with developmental delay, autism, and response to folinic acid. *Neurology*.

[46] Jaeken J, Saudubray J, vanden Berghe G, Walter J (2012). Disorders of serine and proline metabolism. *Inborn Metabolic Diseases: Diagnosis and Treatment*.

[47] Tabatabaie L, Klomp LW, Berger R, de Koning TJ (2010). l-Serine synthesis in the central nervous system: a review on serine deficiency disorders. *Molecular Genetics and Metabolism*.

[48] Koning TJD, Klomp LWJ, Oppen ACCV (2004). Prenatal and early postnatal treatment in 3-phosphoglycerate-dehydrogenase deficiency. *The Lancet*.

[49] De Koning TJ, Jaeken J, Pineda M, Van Maldergem L, Poll-The BT, Van der Knaap MS (2000). Hypomyelination and reversible white matter attenuation in 3-phosphoglycerate dehydrogenase deficiency. *Neuropediatrics*.

[50] Shimomura K, Hörster F, de Wet H (2007). A novel mutation causing DEND syndrome: a treatable channelopathy of pancreas and brain. *Neurology*.

[51] Gloyn AL, Pearson ER, Antcliff JF (2004). Activating mutations in the gene encoding the ATP-sensitive potassium-channel subunit Kir6.2 and permanent neonatal diabetes. *The New England Journal of Medicine*.

[52] Proks P, Arnold AL, Bruining J (2006). A heterozygous activating mutation in the sulphonylurea receptor SUR1 (ABCC8) causes neonatal diabetes. *Human Molecular Genetics*.

[53] Ashcroft FM (2007). ATP-sensitive K^+^ channels and disease: from molecule to malady. *American Journal of Physiology*.

[54] Gurgel LC, Crispim F, Noffs MHS, Belzunces E, Rahal MA, Moisés RS (2007). Sulfonylrea treatment in permanent neonatal diabetes due to G53D mutation in the KCNJ11 gene. *Diabetes Care*.

[55] Mlynarski W, Tarasov AI, Gach A (2007). Sulfonylurea improves CNS function in a case of intermediate DEND syndrome caused by a mutation in KCNJ11. *Nature Clinical Practice Neurology*.

[56] Sperling MA, Menon RK (1999). Hyperinsulinemic hypoglycemia of infancy: recent insights into ATP-sensitive potassium channels, sulfonylurea receptors, molecular mechanisms, and treatment. *Endocrinology and Metabolism Clinics of North America*.

[57] Bahi-Buisson N, Roze E, Dionisi C (2008). Neurological aspects of hyperinsulinism-hyperammonaemia syndrome. *Developmental Medicine and Child Neurology*.

[58] Errazquin FP, Fernández JS, Martín GG, Muñoz MIC, Acebal MR (2011). Hyperinsulinism and hyperammonaemia syndrome and severe myoclonic epilepsy of infancy. *Neurologia*.

[59] Bahi-Buisson N, El Sabbagh S, Soufflet C (2008). Myoclonic absence epilepsy with photosensitivity and a gain of function mutation in glutamate dehydrogenase. *Seizure*.

[60] Palladino AA, Stanley CA (2010). The hyperinsulinism/hyperammonemia syndrome. *Reviews in Endocrine and Metabolic Disorders*.

[61] Brockmann K (2009). The expanding phenotype of GLUT1-deficiency syndrome. *Brain and Development*.

[62] De Vivo DC, Trifiletti RR, Jacobson RI, Ronen GM, Behmand RA, Harik SI (1991). Defective glucose transport across the blood-brain barrier as a cause of persistent hypoglycorrhachia, seizures, and developmental delay. *The New England Journal of Medicine*.

[63] Rotstein M, Engelstad K, Yang H (2010). Glut1 deficiency: inheritance pattern determined by haploinsufficiency. *Annals of Neurology*.

[64] Wang D, Pascual JM, De Vivo D, Pagon RA, Bird TD, Dolar CR (2009). Glucose transporter type 1 deficiency syndrome. *GeneReviews*.

[65] Mills PB, Struys E, Jakobs C (2006). Mutations in antiquitin in individuals with pyridoxine-dependent seizures. *Nature Medicine*.

[66] Basura GJ, Hagland SP, Wiltse AM, Gospe SM (2009). Clinical features and the management of pyridoxine-dependent and pyridoxine-responsive seizures: review of 63 North American cases submitted to a patient registry. *European Journal of Pediatrics*.

[67] Gallagher RC, Van Hove JLK, Scharer G (2009). Folinic acid-responsive seizures are identical to pyridoxine-dependent epilepsy. *Annals of Neurology*.

[68] Mills PB, Footitt EJ, Mills KA (2010). Genotypic and phenotypic spectrum of pyridoxine-dependent epilepsy (ALDH7A1 deficiency). *Brain*.

[69] Pearl PL, Gospe SM (2007). Pyridoxal phosphate dependency, a newly recognized treatable catastrophic epileptic encephalopathy. *Journal of Inherited Metabolic Disease*.

[70] Baxter P (2010). Recent insights into pre- and postnatal pyridoxal phosphate deficiency, a treatable metabolic encephalopathy. *Developmental Medicine and Child Neurology*.

[71] Mills PB, Surtees RAH, Champion MP (2005). Neonatal epileptic encephalopathy caused by mutations in the PNPO gene encoding pyridox(am)ine 5'-phosphate oxidase. *Human Molecular Genetics*.

[72] Summar ML, Dobbelaere D, Brusilow S, Lee B (2008). Diagnosis, symptoms, frequency and mortality of 260 patients with urea cycle disorders from a 21-year, multicentre study of acute hyperammonaemic episodes. *Acta Paediatrica*.

[73] Summar ML, Pagon RA, Bird TD, Dolan CR (2005). Urea cycle disorders overview. *GeneReviews*.

[74] Dealberto MJCC, Sarazin FFA (2008). Valproate-induced hyperammonemic encephalopathy without cognitive sequelae: a case report in the psychiatric setting. *Journal of Neuropsychiatry and Clinical Neurosciences*.

[75] van de Kamp JM, Mancini GMS, Pouwels PJW (2011). Clinical features and X-inactivation in females heterozygous for creatine transporter defect. *Clinical Genetics*.

[76] Nasrallah F, Feki M, Kaabachi N (2010). Creatine and creatine deficiency syndromes: biochemical and clinical aspects. *Pediatric Neurology*.

[77] Fons C, Sempere A, Sanmartí FX (2009). Epilepsy spectrum in cerebral creatine transporter deficiency: letters/commentary. *Epilepsia*.

[78] Leuzzi V (2002). Inborn errors of creatine metabolism and epilepsy: clinical features, diagnosis, and treatment. *Journal of Child Neurology*.

[79] Verhoeven NM, Salomons GS, Jakobs C (2005). Laboratory diagnosis of defects of creatine biosynthesis and transport. *Clinica Chimica Acta*.

[80] Hamosh A, Johnston MV, Sciver CR, Beaudet AL, Sly WS (2001). Nonketotic hyperglycinemia. *The Metabolic and Molecular Bases of Inherited Disease*.

[81] Hoover-Fong JE, Shah S, van Hove JLK, Applegarth D, Toone J, Hamosh A (2004). Natural history of nonketotic hyperglycinemia in 65 patients. *Neurology*.

[82] Rossi S, Daniele I, Bastrenta P, Mastrangelo M, Lista G (2009). Early myoclonic encephalopathy and nonketotic hyperglycinemia. *Pediatric Neurology*.

[83] Bonioli E (1996). Combined deficiency of xanthine oxidase and sulphite oxidase due to a deficiency of molybdenum cofactor. *Journal of Inherited Metabolic Disease*.

[84] Johnson JL, Duran M, Sciver CR, Beaudet AL, Sly WS (2001). Molybdenum cofactor deficiency and isolated sulfite oxidase deficiency. *The Metabolic and Molecular Bases of Inherited Disease*.

[85] Van Gennip AH, Abeling NGGM, Stroomer AEM, Overmars H, Bakker HD (1994). The detection of molybdenum cofactor deficiency: clinical symptomatology and urinary metabolite profile. *Journal of Inherited Metabolic Disease*.

[86] Sie SD, de Jonge RC, Blom HJ (2010). Chronological changes of the amplitude-integrated EEG in a neonate with molybdenum cofactor deficiency. *Journal of Inherited Metabolic Disease*.

[87] Vijayakumar K, Gunny R, Grunewald S (2011). Clinical neuroimaging features and outcome in molybdenum cofactor deficiency. *Pediatric Neurology*.

[88] Prasad AN, Rupar CA, Prasad C (2011). Methylenetetrahydrofolate reductase (MTHFR) deficiency and infantile epilepsy. *Brain and Development*.

[89] Ueland PM, Holm PI, Hustad S (2005). Betaine: a key modulator of one-carbon metabolism and homocysteine status. *Clinical Chemistry and Laboratory Medicine*.

[90] Ciardo F, Salerno C, Curatolo P (2001). Neurologic aspects of adenylosuccinate lyase deficiency. *Journal of Child Neurology*.

[91] Mizuno T (1986). Long-term follow-up of ten patients with Lesch-Nyhan syndrome. *Neuropediatrics*.

[92] Sass JK, Itabashi HH, Dexter RA (1965). Juvenile gout with brain involvement. *Archives of Neurology*.

[93] Vajsar J, Schachter H (2006). Walker-Warburg syndrome. *Orphanet Journal of Rare Diseases*.

[94] Warburg M (1978). Hydrocephaly, congenital retinal nonattachment, and congenital falciform fold. *American Journal of Ophthalmology*.

[95] Fukuyama Y, Osawa M, Suzuki H (1981). Congenital progressive muscular dystrophy of the Fukuyama type—clinical, genetic and pathological considerations. *Brain and Development*.

[96] Mercuri E, Topaloglu H, Brockington M (2006). Spectrum of brain changes in patients with congenital muscular dystrophy and FKRP gene mutations. *Archives of Neurology*.

[97] Jalanko A, Braulke T (2009). Neuronal ceroid lipofuscinoses. *Biochimica et Biophysica Acta*.

[98] Mole SE, Williams RE, Pagon RA, Bird TC, Dolan CR (2005). Neuronal ceroid-lipofuscinoses. *GeneReviews*.

[99] Santavuori P, Vanhanen SL, Autti T (2001). Clinical and neuroradiological diagnostic aspects of neuronal ceroid lipofuscinoses disorders. *European Journal of Paediatric Neurology A*.

[100] Sidransky E (2004). Gaucher disease: complexity in a “simple” disorder. *Molecular Genetics and Metabolism*.

[101] Tylki-Szymańska A, Vellodi A, El-Beshlawy A, Cole JA, Kolodny E (2010). Neuronopathic Gaucher disease: demographic and clinical features of 131 patients enrolled in the International Collaborative Gaucher Group Neurological Outcomes Subregistry. *Journal of Inherited Metabolic Disease*.

[102] Maegawa GHB, Stockley T, Tropak M (2006). The natural history of juvenile or subacute GM2 gangliosidosis: 21 new cases and literature review of 134 previously reported. *Pediatrics*.

[103] Gressens P (2006). Pathogenesis of migration disorders. *Current Opinion in Neurology*.

[104] Raymond GV (2001). Peroxisomal disorders. *Current Opinion in Neurology*.

[105] Shimozawa N (2007). Molecular and clinical aspects of peroxisomal diseases. *Journal of Inherited Metabolic Disease*.

[106] White AL, Modaff P, Holland-Morris F, Pauli RM (2003). Natural history of rhizomelic chondrodysplasia punctata. *American Journal of Medical Genetics A*.

[107] Prust M, Wang J, Morizono H (2011). GFAP mutations, age at onset, and clinical subtypes in Alexander disease. *Neurology*.

[108] Papetti L, Parisi P, Leuzzi V (2012). Metabolic epilepsy: an update. *Brain and Development*.

